# Tyrosine-kinase expression profiles in human gastric cancer cell lines and their modulations with retinoic acids

**DOI:** 10.1038/sj.bjc.6600821

**Published:** 2003-04-01

**Authors:** H-W Kao, H-C Chen, C-W Wu, W-C Lin

**Affiliations:** 1 Institute of Biomedical Sciences, Academia Sinica, Taipei 115, Taiwan, ROC; 2 Taigen Biotechnology, 7F, No. 138 Shin Ming Road, Taipei 114, ROC; 3 Department of Surgery, Taipei-Veterans General Hospital, National Yang-Ming University, Taipei 112, Taiwan, ROC; 4 Institute of Biotechnology in Medicine, National Yang-Ming University, Taipei 112, Taiwan, ROC; 5 Graduate Institute of Basic Medical Sciences, Chang Gung University, Tao-Yuan 333, Taiwan, ROC

**Keywords:** protein-tyrosine kinase, retinoic acid, gene expression profiles

## Abstract

Many protein tyrosine kinases are key regulators involved in cellular growth, differentiation, development, apoptosis and signal transduction pathways. Obtaining a comprehensive tyrosine-kinase expression profile in tumour cells is essential to learning more about their oncogenic potentials and responses to various chemotherapeutic reagents – such as retinoic acid, which has been shown to suppress the growth of gastric cancer cells and modulate gene expression. Expression of tyrosine kinases in retionic acid-treated cancer cells was investigated by reverse trancriptase–polymerase chain reaction (RT–PCR) and a novel restriction analysis of gene expression (RAGE) display technique. We first established comprehensive tyrosine-kinase profiles in different human gastric cancer cell lines. In cells treated with 9-*cis*-retinoic acid or all-*trans*-retinoic acid, we found that two PTKs (*Eph* and *Hek5*) appeared to be upregulated. In the present study, we demonstrate an efficient and simple RAGE approach for examining tyrosine kinases' expression in tumour cells and their alterations following drug treatments.

It is estimated that there are 1000–2000 different protein kinases in the human genome (Hunter and Cooper, 1985; Robinson *et al*, 2000). Less than 10% of all kinases are protein tyrosine kinases (PTK), but many tyrosine kinases are involved in growth signalling, and alterations of these kinases often result in cellular transformation (Hunter and Cooper, 1985). Many PTK genes are proto-oncogenes and some of them are associated with human cancer progression, including *erb*B2/neu in breast cancer and m*et* in gastric cancer (Kameda *et al*, 1990; Kuniyasu *et al*, 1992). These genes are highly conserved from nematode to human and are involved in important biological functions such as growth, differentiation, development, apoptosis and signal transduction. On the basis of their cellular localisation, these PTKs can be further classified as receptor-type or nonreceptor-type PTK and dual kinases that can phosphorylate both tyrosine and serine/threonine residues. Since PTKs share significant homologies in their kinase catalytic domain, degenerated polymerase chain reaction (PCR) primers can be designed according to the amino-acid sequence submotifs of the kinase catalytic domain (Robinson *et al*, 1996; Lin *et al*, 1998). In previous studies, we identified 25–35 different PTK genes expressed in cells by simple reverse trancriptase–polymerase chain reaction (RT–PCR) reaction (Lin *et al*, 1998, 1999; Wu *et al*, 2000). Since then, we implemented an improved restriction analysis of gene expression (RAGE) profiling approach, developed at Dr Hsing-Jien Kung's laboratory (Robinson *et al*, 1996), which utilises restriction enzyme digestion and gel electrophoresis for quick and efficient kinase profiling (manuscript in preparation). Recently, this profiling method has been used to generate expression profiles of human breast cancer cell lines (Meric *et al*, 2002) and also identified *axl* receptor tyrosine kinase as one of the adenovirus E1A target genes in ovarian cancer cells (Lee *et al*, 1999).

Retinoic acid (RA) and its metabolites, the retinoids, are required for differentiation and tissue maintenance. Deficiencies in the metabolism of retinoids are associated with severe defects of vertebrate embryonic development (Gudas, 1992; Chambon, 1994). This reagent has been used in the treatment of acute promyelocytic leukaemia with good success (Huang *et al*, 1988), and recently it was shown to suppress the growth of gastric cancer cell lines (Shyu *et al*, 1995; Naka *et al*, 1997). The RA signalling pathway involves specific high-affinity receptors, which belong to ligand–inducible transcription factors (Chambon, 1996). The RA ligand-receptor complexes modulate target genes' expression by binding to the retinoid-responsive elements in the promoter regions. Among PTKs, c-erbB2/neu promoter contains retinoid-responsive elements, which are weakly modulated by the addition of RA in the medium (Hudson *et al*, 1990). All-*trans*-RA was recently shown to enhance mRNA expression of *c-fms* receptor tyrosine kinase in human breast carcinoma cells (Sapi *et al*, 1999). Extensive research on the expression of PTKs and RA modulation is lacking and details regarding the mechanisms involved in the RA-regulated growth inhibition of gastric cancer cells are not known. In the present study, we examined the overall PTK expression profiles in gastric cancer cells treated with 9-*cis*-RA or all-*trans*-RA. We found that expressions of *eph* (*ephA1*) and *hek5* (*ephB2*) were upregulated following treatment in several gastric cancer cell lines.

## MATERIALS AND METHODS

### RT–PCR amplification and RAGE profiling of human tissues

Poly(A) selected mRNA were purchased from Clontech, including eight adult tissues (brain, kidney, liver, lung, pancreas, placenta, small intestine and stomach) and four foetal tissues (foetal brain, foetal kidney, foetal liver and foetal lung). Reverse transcription was carried out with a cDNA synthesis kit (Boehringer Mannheim, Mannheim, Germany). The PCR primers are derived from the conserved motifs DFG and DVW of tyrosine-kinase catalytic domain as described (Meric *et al*, 2002). Several pairs of degenerated PCR primers were designed, which would expect a PCR product of 150–170 bp range. In order to identify the restriction-digested fragments, the 5′-sense primers were radio-labelled with ^33^P by polynucleotide kinase in this RAGE method. The PCR reactions were conducted at 42° annealing temperature for five cycles and then at 55° for 25 cycles with an Accugen 9600 PCR thermocycler and *Taq* polymerase (GIBCO-BRL, Rockville, MD, USA). The final PCR products at 170 bp fragment products were separated and eluted from agarose gels. An equal amount (20 000 c.p.m.) of the eluted PTK amplicon was then digested with respective restriction enzymes (New England BioLabs, Beverly, MA, USA) and analysed with denaturing 7% sequencing polyacrylamide gels. Following electrophoresis, the gel was dried and exposed to an X-ray film or processed by a Fuji BAS 6000 phosphoimager (Fuji photo film, Tokyo, Japan). A sequen-cing reaction product (^35^S-label, and T-track only) with a predetermined sequence template was used as a standard for fragment size. We have pre-established a restriction fragment database of human PTKs digested with 18 different restriction enzymes, and individual tyrosine kinase was identified based on its respective characteristic restriction fragment sizes on the exposed X-ray films or in the phosphoimager-processed files.

### Gastric cancer cell lines and RA treatment

Six human gastric cancer cell lines were used in this study, including HR, AGS, KATO III, NUGC (NUGC-3), TSGH (TSGH9201) and SC-M1 (Lin *et al*, 1998). All cell lines, except HR, were kindly provided by Dr Chin-Wen Chi at Taipei-Veterans General Hospital, Taiwan. These cells were cultured in RPMI 1640 or DMEM culture medium supplemented with 10% foetal calf serum, 2 mM glutamine, 100 U ml^−1^ penicillin, and 100 *μ*g ml^−1^ streptomycin in 5% CO_2_/95% air at 37°C. 9-*cis*-Retionic acid and all-*trans*-RA were purchased from Sigma (St. Louis, MO, USA). Retinoic acid was dissolved in dimethyl sulphfoxide (DMSO) under subdued light in a tissue culture hood. Three cell lines were selected for RA treatment based on an earlier report of their sensitivity to RA (Shyu *et al*, 1995) as well as their tolerance to serum-free culture conditions. Since serum could contain retinoids and other steroid hormones, serum-free culture conditions were preferable to reduce the background. Cells were seeded overnight at a density of 1 × 10^6^ cells per 100-mm tissue culture plates. The cells were then washed with serum-free medium and maintained in serum-free culture medium with the presence or absence of RA for 36–48 h. The final concentration of RA used in the medium was 10^−6^–10^−8^ M and the concentration of DMSO in control groups was 0.1%. The growth response of RA-treated cells was measured by an MTT assay (Yasumura *et al*, 1994).

### RAGE PTK profiling of human gastric cancer cells

Total RNA was extracted from exponentially growing gastric cancer cells by TRIzol reagent (GIBCO-BRL, Rockville, MD, USA), and the RNA pellets were washed several times with 70% ethanol, dried and resuspended with RNase-free water. Reverse transcription was carried out and RAGE PTK profiles were performed as described above.

### RT–PCR expression analysis of selected kinases

Total RNA samples from various gastric cancer cell lines were used in reverse transcription reactions with oligo (dT)_15_ primers as described above. The resulting cDNA was subjected to PCR reaction by using gene-specific primers. The PCR was conducted in 25 *μ*l reactions each containing 200 *μ*M dNTP, 1.25 mM MgCl_2_ and 800 nM of the specific primer for 35 cycles at 58°C annealing temperature for 30 s, 72°C for 30 s and 94°C for 30 s. The primer sets for the kinases used in this study were:


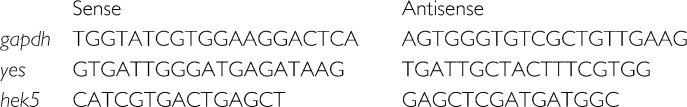


The expected sizes of PCR products were 371 bp for *GAPDH*, 318 bp for *yes* and 420 bp for *hek5*. The final products were analysed in 2% agarose gel, visualised by ethidium bromide staining and recorded with the Alpha Innotech IS-500 gel documentation system.

### Western blot analysis of yes kinase

Gastric cancer cell lines were incubated in NP-40 lysis buffer containing 50 mM Tris pH 7.4,. 150 mM NaCl, 1 mM EGTA, 1% NP-40, 0.25% SDS, 1 mM sodium vanadate, 1 *μ*g ml^−1^ protease inhibitors, 200 *μ*g ml^−1^ chymostatin and 1 mM PMSF for 30 min at 4°C. Cell lysates were then centrifuged at 14 000 rpm for 10 min and supernatants were harvested. A measure of 100 *μ*g of lysates was boiled and electrophoresed in 7.5% SDS–polyacrylamide gels under reducing conditions. The separated proteins were then electrophoretically transferred to a PVDF membrane (Immoblin-P, Millipore, Bedford, MA, USA). Following blocking by blocking solution of 10% nonfat dried milk for 1 h at room temperature, the membrane was blotted with anti-yes-specific antibody (Santa Cruz Biotech, Santa Cruz, CA, USA) or anti-*β*-actin antibody (Santa Cruz Biotech, Santa Cruz, CA, USA) at room temperature for 1 h. Following washing with Tris-buffered saline with 0.05% Tween-20 and incubation with horseradish peroxidase-conjugated secondary antibodies, protein bands were detected by the enhanced chemiluminescence method (Amersham Life Science, Piscataway, NJ, USA).

## RESULTS

**Figure 1:**
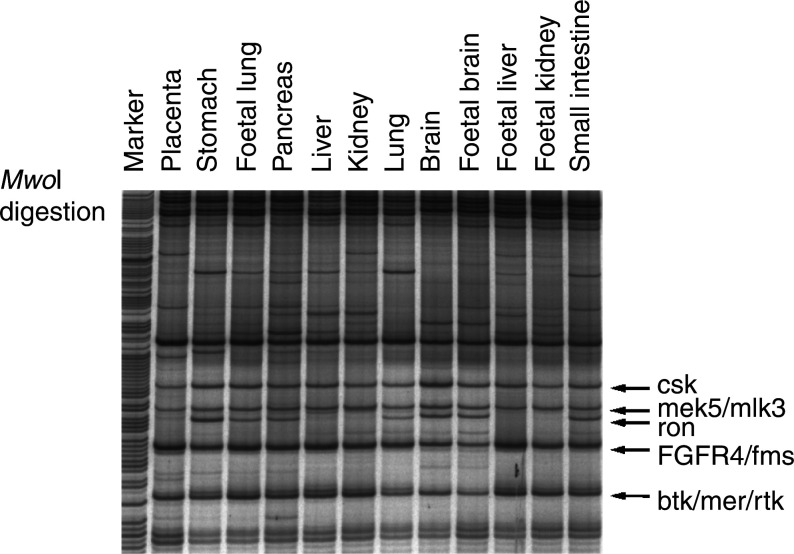
RAGE PTK profiling of different human tissues. Poly(A) mRNA obtained from Clontech was amplified by RT–PCR as described in Materials and Methods. The 150–170 bp amplicon was purified and used for *Mwo*I digestion. The completed digested products were separated by a denaturing sequencing gel. Specific PTKs were identified by the digested fragment sizes as indicated.

By using degenerated primers from submotifs VII and IX of the kinase domain as well as RT–PCR, we were able to easily amplify PTK genes expressed in cells. In addition to the previous ‘cloning and sequencing’ method, we utilised an improved RAGE method to provide a more efficient, economical and expeditious tyrosine-kinase profiling approach. We first established the PTK RAGE protocol in our laboratory by using a panel of human tissues. Samples from 12 tissues could be displayed on a single sequencing gel; thus we could effectively screen all known human PTKs in a short period of time. As shown in Figure 1

, the amplified PTK products was digested with *Mwo*I restriction enzyme and separated on a sequencing gel. In Figure 1, several PTKs could be identified by their specific restriction fragment sizes. As examples, the 73 bp fragment represented *csk* kinase gene; 67 bp fragment for *mek5/mlk3*; 65 bp fragment for *ron* kinase; 59 bp fragment for *FGFR4/fms*; 50 bp fragment for *btk/mer/rtk* kinase genes. In many cases, several different kinases could be represented by similar size restriction fragments, and alternative restriction enzymes would be necessary to identify single-gene-specific restriction fragments. It was calculated that just 15–20 restriction enzymes (four-base or five-base hitters) were required to cover all known PTKs. Among these kinases in Figure 1, *ron* kinase expression was found in selected tissues only (brain, lung, pancreas, small intestine, stomach, foetal brain and foetal lung). This finding correlated with the reported *in situ* hybridisation and Northern blot data (Quantin *et al*, 1995), which validated our PTK profiles. By eliminating cloning and sequencing steps in previous profiling approaches (Lin *et al*, 1998) and adopting end-labelled PCR primers, this RAGE method is more quantitative as the intensity of each PTK fragments could reflect the PTK molecules expressed in cells.

This approach enabled us to establish comprehensive PTK expression profiles from a large number of samples in a single experiment, and thus we were able to generate comprehensive PTK profiles from six different human gastric cancer cell lines in a very limited amount of time. Two cell lines (AGS and SC-M1) were derived from primary tumours and others (HR, Kato III, NUGC, TSGH) were derived from metastatic tumours from various organs. With this PTK profile information, we hoped to learn more about the biological significance of particular PTKs in the oncogenesis of these human gastric cancer cells.

**Figure 2:**
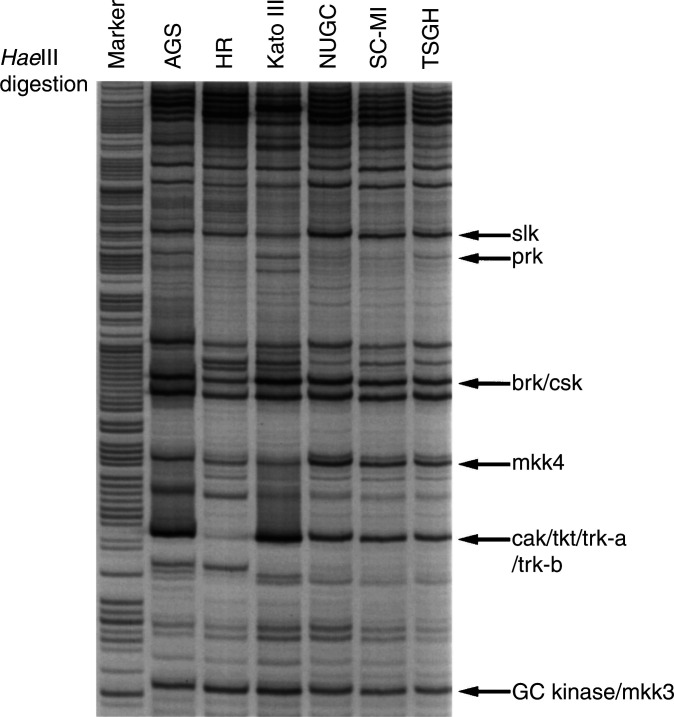
RAGE PTK profiling of different human gastric cancer cell lines. Total RNA obtained from six human gastric cancer cell lines (AGS, HR, Kato III, NUGC, SC-M1 and TSGH) was amplified by RT–PCR as described in Materials and Methods. The 150–170 bp amplicon was purified and used for *Hae*III digestion. The completed digestion products were separated by a denaturing sequencing gel. Specific PTKs were identified by their digested fragment sizes as indicated.

**Figure 3:**
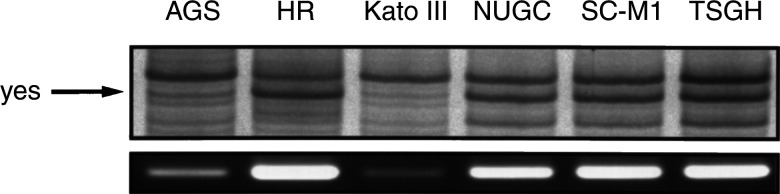
RAGE profile and RT–PCR pattern of *yes* tyrosine kinase in human gastric cancer cell lines. Upper panel: RAGE expression profile of the *yes* kinase gene. Lower panel: *yes* kinase expression detected by RT–PCR method with specific primers.

**Figure 4:**
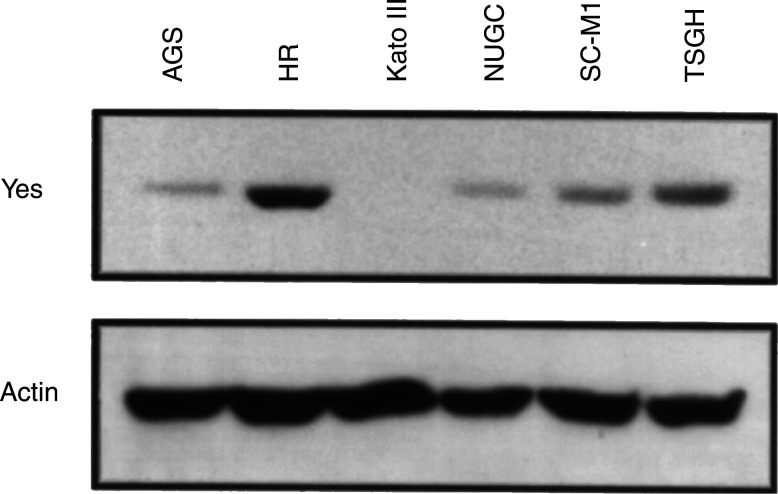
Immunoblot showing the expression of yes tyrosine kinase in human gastric cancer cell lines. An equal amount of cell lysate from human gastric cancer cell lines was resolved by SDS–PAGE. After electrophoresis, proteins were transferred to a PVDF nylon membrane and then probed with anti-yes antibody. The anti-*β*-actin antibody was used a control.

As shown in Figure 2

, the amplified PTK products were digested with *Hae* III restriction enzyme and displayed on a sequencing gel. Samples from six human gastric cancer cell lines could be displayed on a single sequencing gel; thus, we could effectively screen all known human PTKs with only a few gel displays. In Figure 2, several PTKs could be identified by their specific restriction fragment sizes. Alternative restriction enzymes would be necessary to identify all PTKs according to their unique specific restriction fragment. This RAGE method is more quantitative as the radioactive isotope is labelled on the primers. Therefore, we could measure PTK expression levels by enumerating radioactivities of each fragment. In Figure 3

, we correlated the expression of the yes PTK gene by specific primers. The RT–PCR results reflected the RAGE profile. AGS and Kato III showed a lower level of yes expression in both assays. In order to validate the protein expression level, Western blot analysis was performed with yes PTK-specific antibody. As shown in Figure 4

, overexpression of yes tyrosine-kinase protein is evident. The *yes* PTK expression pattern is similar in the mRNA and protein level in this study (Figure 3 and Figure 4).

Figure 5

**Figure 5:**
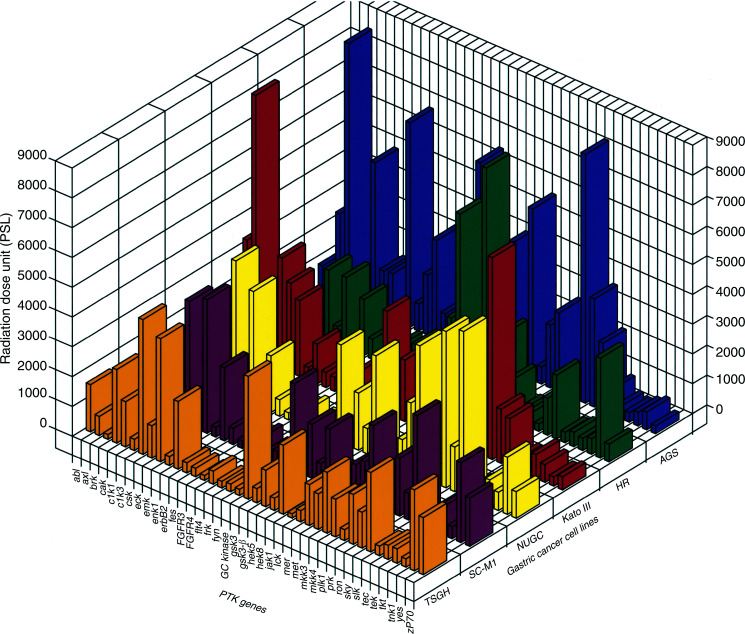
PTK expression profiles of six human gastric cancer cell lines. Total RNA was isolated from exponentially growing human gastric cancer cell lines (AGS, HR, Kato III, NUGC, SC-M1 and TSGH) and used for RAGE analysis as described in Materials and Methods. A total of 39 different human kinases were identified and the expression level of each kinase was determined using a Fuji BAS phosphoimager and are represented by their radiation dose units (PSL).

summarises the complete PTK profiles in these gastric cancer cell lines, and a total of 39 protein kinases were identified. Each cell line generated a unique and distinct PTK expression profile. This valuable information can now be used as an overall PTK expression reference database and serve as PTK fingerprints of different gastric cancer cell lines. Four cell lines of Asian origins (Japan and Taiwan) presented surprisingly similar profiles, although Kato III had an enhanced expression on brk and cak kinases and a lower expression level on slk. On the other hand, two PTKs (hek8 and mer) expressed at a higher level in AGS and HR cells. There were also significant variations between cell lines, including a low expression level of ron in HR cells. This information allows us to discern the tyrosine-kinase expression pattern in a particular cell line and to select proper cells for tyrosine-kinase functional studies. For example, AGS might not be suitable for erbB2/neu transfection experiments, since it has the highest expression of that particular tyrosine kinase (Figure 5). On the same token, HR cells would be the preferable choice for studying cak kinase because of its extremely low expression level (Figure 2 and Figure 5). There was a small number of different PTKs identified between previous cloning experiments using HR cells and the new RAGE profile of HR cells. This could largely be attributed to the different degenerated primers used in the two types of experiments.

Upregulation of some PTKs, such as c-erbB2/neu, confers resistance to chemotherapeutic reagents (Yu *et al*, 1996). It is important to know the PTKs modulated by chemotherapeutic reagents and possible resistant mechanisms involved in cancer cells. This RAGE-based PTK profiling allowed us to quickly screen tyrosine-kinase genes modulated in cells treated with various reagents. We first selected RA for our studies, since it directly modulates gene expressions following its binding to RAR or RXR receptors. In addition, RA was shown to suppress gastric cancer cell growth *in vitro* (Shyu *et al*, 1995; Naka *et al*, 1997). We used both 9-*cis*-RA and all-*trans*-RA and three RA responsive cell lines (NUGC, SC-M1, TSGH) in this study. By MTT assay, we had determined that growth of these gastric cancer cells was inhibited by 10^−8^–10^−6^ M in both all-*trans*-RA and 9-*cis*-RA. Cell death was observed after 5–7 day treatment with RA. Since it was shown that most RA responsive genes are activated by 24 h posttreatment (Bouillet *et al*, 1995), we therefore selected an optimum condition of 10^−6^ M under serum-free conditions and a 36-h treatment schedule for mRNA extractions, at which point most cells (>80%) were still viable.

**Figure 6:**
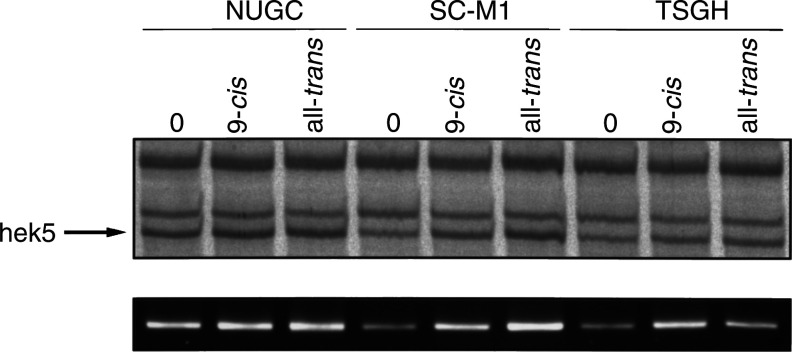
Modulation of *hek5* kinase in three gastric cancer cell lines treated with 9-*cis*-RA and all-*trans*-RA. NUGC, SC-M1 and TSGH cells were treated with 10^−6^ M 9-*cis*-RA or 10^−6^ M all-*trans*-RA for 36 h. Total RNA was extracted and used for PTK RAGE analysis. The RAGE profile of *hek5* expression is shown in the upper panel. In the lower panel, expression of *hek5* is demonstrated by RT–PCR using *hek5*-specific primers.

According to PTK RAGE profile analysis, there were several kinases upregulated by 9-*cis*-RA and all-*trans*-RA in all cells, including *eph*, *hek5* and several novel kinases. In Figure 6

, *hek5* RAGE profiles are shown in the upper panel. To verify the expression of *hek5*, we also performed RT–PCR experiments with *hek5*-specific primers (Figure 6, lower panel). Similar results were obtained with the *eph* kinase (not shown). There was a notable increase in *hek5* expression level in SC-M1 and TSGH cells (Figure 6). Both *eph* and *hek5* belong to the *eph* tyrosine-kinase subgroup, which is the largest subgroup of tyrosine kinases and which plays important roles in development (Zisch and Pasquale, 1997). *Hek5* expression was previously shown to be elevated in human gastric cancer tissues (Kiyokawa *et al*, 1994). However, the present study is the first to show the modulation of *eph* and *hek5* by RA treatment in human gastric cancer cell lines. In a reported RA-resistant cell line (AGS) (Shyu *et al*, 1995), basal levels of *hek5* and *c-erbB2*/*neu* expression were markedly elevated in comparison with those of RA-sensitive cell lines (Figure 6). Since amplification of *c-erbB2/neu* could lead to drug-resistant phenotypes in tumour cells (Yu *et al*, 1996), it would be interesting to investigate if *hek5* kinase is also involved in such mechanisms.

## DISCUSSION

We have developed a simple, economical and efficient method for generating comprehensive expression profiles of gene families. With this technique, we were able to effectively generate PTK expression profiles of human gastric cancer cell lines. These profiles serve as fingerprints for each tumour cell line and are extremely valuable in correlating various biological properties of these tumour cells *in vitro* and *in vivo*. Thus, we should be able to learn more about the roles of PTKs in tumour initiation and progression, and develop better therapeutic reagents or strategies.

Retinoic acid is the active derivative of vitamin A, and interacts with two classes of receptors (RARs and RXRs). Both RARs and RXRs are ligand-dependent transcription factor receptors (Chambon, 1996). RAR and RXR families each posseses three receptor subtypes (*α*, *β* and *γ*), which have been demonstrated to be differentially expressed in various tissues during the mouse embryo development (Ruberte *et al*, 1991). RARs are activated by all-*trans*-RA acid and 9-*cis*-RA, whereas RXRs are only activated by 9-*cis*-RA (Heyman *et al*, 1992). RA regulates the growth and differentiation of many different cell types, and plays an important role in development (De Luca, 1991). Many genes are known to be modulated by RA treatment, such as Stra genes (Stra1–13) (Bouillet *et al*, 1995; Boudjelal *et al*, 1997), midkine (Muramatsu *et al*, 1993), ICAM-1 (Cilenti *et al*, 1995), STAT1 (Kolla *et al*, 1996), IL-8 (Harant *et al*, 1996) and IL-2 receptor alpha subunit (Bhatti and Sidell, 1994). Among signal transduction molecules, c-erbB2 promoter activity and X17C (a novel MAP kinase phosphatase) were found to be upregulated by RA (Hudson *et al*, 1990; Mason *et al*, 1996). RAR and RXR can also be phosphorylated by protein kinases (Tahayato *et al*, 1993). In a previous report on RARs' and RXRs' Northern mRNA expression level of gastric cancer cell lines (Shyu *et al*, 1995), enhanced expression of RAR*α*/RAR*γ* was observed in SC-M1 cells and enhanced RAR*β* level was found in TSGH cells. Both RA-sensitive cell lines (SC-M1 and TSGH) had also increased RXR*α* expression, but only TSGH had higher RXR*β* expression transcript. The authors implicated that RAR*β* and RXR*β* may not be related to the RA-mediated growth inhibitory effect in these sensitive cells, while RAR*γ* may play an important role in these cells (Shyu *et al*, 1995). Nevertheless, hypermethylation of the RAR*β* promoter resulted in the loss of RAR*β*, and *nm23*-H1 genes were reported in diffuse-type gastric cancers (Oue *et al*, 2002). With our PTK profiling technique, we identified two PTKs (*eph* and *hek5*) that also responded to RA treatment in RA-sensitive gastric cancer cell lines. The actual roles of these kinases in RA-mediated growth inhibition need to be further examined. Owing to the complex homo- or hetero-dimer interactions between RARs and RXRs, it is difficult to dissect the detail mechanisms of RARs' and RXRs' activation, not to mention mechanisms involving other retinoic/retinol binding proteins within the cytoplasm. It is also important to identify those novel kinases in RAGE profiles that are regulated by RA treatments. This could be achieved by direct cloning of restriction fragments from gels or by bioinformatic databases interrogations. We did not observe the expected upregulation of *c-erbB2/neu* PTK in the gastric cancer cells examined.

*Eph* receptor tyrosine kinases (including *eph* and *hek5*) and their ligands (*ephrins*) play important functions during embryogenesis, especially in neuronal tissue growth, migration and differentiation (Zisch and Pasquale, 1997; Mellitzer *et al*, 1999). Overexpressions of *ephs* and *ephrins* are documented in many human cancers including gastrointestinal cancers (reviewed in Nakamoto and Bergemann, 2002). There are more than 14 members of the *eph* PTK family and at least eight different ephrins. Since ephrins are attached to the cell membrane by either glycosylphosphatidyl-inosital-anchor or a single transmembrane domain, they might act as receptor-like signalling molecules that can be phosphorylated and then transduce signals (Bruckner *et al*, 1997). Autocrine expression of *eph* kinase and ephrins was recently demonstrated in human lung cancer cell lines and tissues (Tang *et al*, 1999). *Ephrin-B1* (also known as lerk-2, stra-1 and cek5-L), which is the ligand for *hek5*, *hek10* and *elk*, is strongly upregulated by RA in mouse P19 embryonal carcinoma cells and D3 embryonic stem cells (Bouillet *et al*, 1995). Since *hek5* is also upregulated in gastric cancer cells following RA treatment, we are now investigating the expression of *ephrin-B1* and *hek5* in detail to determine their functions in human gastric cancer cell growth and in RA-mediated cell growth inhibition. On the other hand, *hek8* (*ephA4*) was downregulated by RA treatment in developing chick limb buds (Patel *et al*, 1996). However, there is no literature report regarding the RARs' or RXRs' involvement in the *eph*/*ephrin* signalling pathway following RA treatment. It is evident that extremely complicated networks exist Q1within the *eph*/*ephrin* as well as the RA/RAR-RXR systems because of the large numbers of gene family members and crossinteractions. This report provides an initial but significant observation on the crosstalk between these two complicated signal networks. Such crosstalk interactions among different signal transduction pathways have been documented recently between IL-6 and EGF pathways in prostate cancer cells (Qiu *et al*, 1998). With this new RAGE profiling approach, we are able to show the potential interactions between *eph* and RA pathways in human gastric cancer cell lines.
